# Oligonucleotides Isolation and Separation—A Review on Adsorbent Selection

**DOI:** 10.3390/ijms23179546

**Published:** 2022-08-23

**Authors:** Sylwia Studzińska, Łukasz Nuckowski, Bogusław Buszewski

**Affiliations:** Department of Environmental Chemistry and Bioanalytics, Faculty of Chemistry, Nicolaus Copernicus University, PL-87-100 Toruń, Poland

**Keywords:** antisense oligonucleotides, impurities, biotransformation products, solid support, polar, hydrophobic, ion-exchange groups

## Abstract

Oligonucleotides have many important applications, including as primers in polymerase chain reactions and probes for DNA sequencing. They are proposed as a diagnostic and prognostic tool for various diseases and therapeutics in antisense therapy. Accordingly, it is necessary to develop liquid chromatography and solid phase extraction methods to separate oligonucleotides and isolate them from biological samples. Many reviews have been written about the determination of these compounds using the separation technique or sample preparation for their isolation. However, presumably, there are no articles that critically review the adsorbents used in liquid chromatography or solid phase extraction. The present publication reviews the literature from the last twenty years related to supports (silica, polymers, magnetic nanoparticles) and their modifications. The discussed issues concern reversed phase (alkyl, aromatic, cholesterol, mixed ligands), ion-exchange (strong and weak ones), polar (silica, polyhydroxy, amide, zwitterionic), and oligonucleotide-based adsorbents.

## 1. Introduction

**Figure 1:**
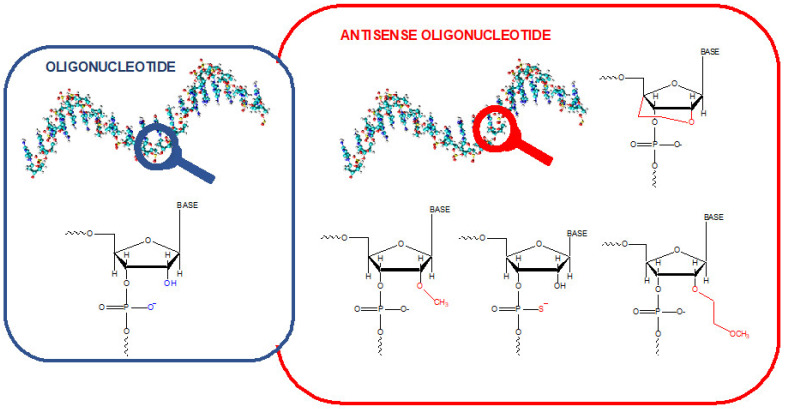
Schematic representation of the difference between the unmodified oligonucleotide and some antisense oligonucleotides.

Oligonucleotides (OGNs) are fragments of nucleic acids, such as intermediate degradation products of full-length DNA and RNA or microRNAs, which regulate processes in biological systems. Their expression may be deregulated when diseases are developed. Therefore, they are proposed as a diagnostic and prognostic tool for various diseases [1,2]. Single-stranded OGNs with any specified sequence can be synthesized in the laboratory. Such molecules have a wide range of applications, including research, disease diagnosis, and therapy. OGNs used as therapeutics have high growth potential. They are used as starters in polymerase chain reactions, allowing for gene expression studies or probes for DNA sequencing, characterization, and tracking nucleic acids in biological systems. Antisense oligonucleotides (ASO) (Figure 1) are chemically modified and can bind to a specific fragment of mRNA and efficiently block protein synthesis [3,4]. Some of them enable the synthesis of an alternative functional or even the correct protein instead of the mutated one. They are clinically tested as potential therapeutics in various diseases [5,6]. Many of them are still at different stages of clinical trials. However, there has been a significant breakthrough in this field in the past seven years. In this time, 14 ASOs have found use in treating several diseases (primarily genetic), such as, e.g., Duchenne Muscular Dystrophy, Spinal Muscular Atrophy, Familial Amyloid Neuropathies, and Acute Hepatic Porphyria [5,6].

The analysis of OGNs is important for impurity determination, degradation, or biotransformation product analysis. Medical utilization of OGNs also requires analysis, e.g., the detection of microRNA concentration changes helps in disease diagnosis, while the quantitative and qualitative determination of ASO is a crucial aspect of clinical studies for their potential application as drugs [1,2,7]. The most commonly used techniques are liquid chromatography (LC) and solid phase extraction (SPE). The first of which is the main technique for a separation of OGN mixtures, while SPE is used due to high OGN recoveries and very good reproducibility of extraction process. Both techniques utilize adsorbents based on interactions between the analyte, liquid phase, and solid phase. Stronger interaction between OGNs and adsorbent surfaces affects stronger retention and, at the same time, may cause higher resolution or extraction efficiency. Thus, an appropriate selection of adsorbents is an essential aspect of OGN separation and extraction. Silica and polymeric-based supports are mainly used during liquid chromatographic analysis of ASO. Similar supports are used during SPE, and magnetic particles have increasing potential here. The surface of these materials is usually chemically modified with various functional groups to ensure specific interactions between OGNs and adsorbents. Due to the type of modification, they are used in different modes. Separations are usually performed by reversed-phase (RP HPLC), ion-exchange (IEC), and hydrophilic interactions liquid chromatography (HILIC). SPE is additionally performed using pure silica in the presence of chaotropic salts or OGNs modified adsorbents. Materials used as stationary phases allow for separation and quality control of impurities.

Moreover, they facilitate the purification of samples contaminated with synthesis by-products. However, due to their complex nature, the separation of OGNs does not always ensure their successful analysis, mostly when studying biological matrices. Thus, they must be correctly prepared, and it is mainly done with SPE [8,9].

**Figure 2:**
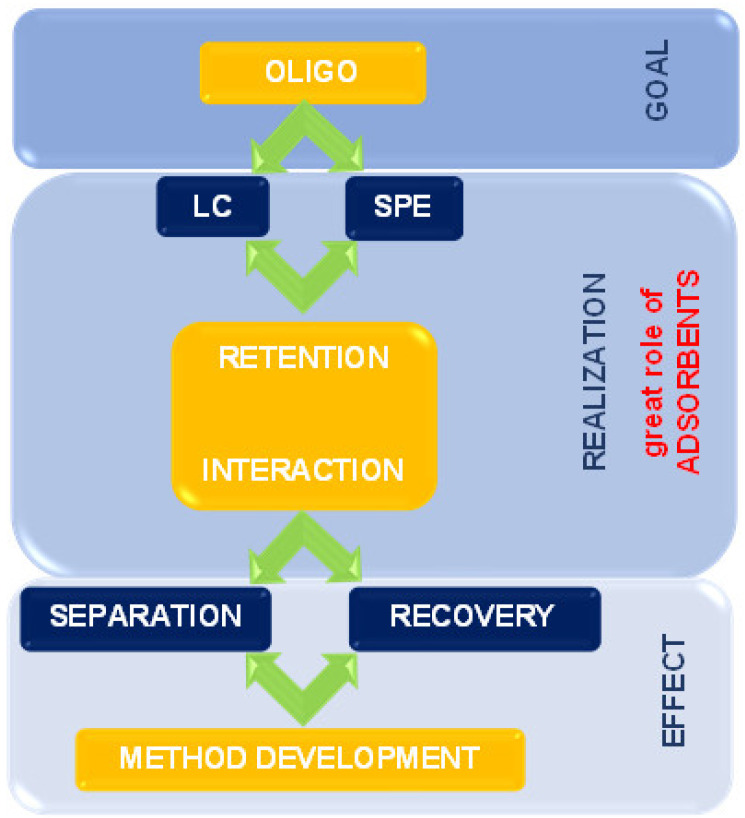
The scheme indicates the place and role of solid materials used in ASO analysis.

The present review summarizes the state-of-the-art supports and adsorbents used for the past twenty years for OGN purification and separation. The issues connected with retention mechanisms and interactions during extraction and chromatographic processes are also discussed. It is essential to apply appropriate solid phases to improve separation ability, selectivity, and extraction efficiency (Figure 2).

## 2. Types of Supports Used in OGNs Purification and Separation 

Generally, two supports are used in OGN separation and extraction, namely silica and polymer-based materials. However, magnetic particles are also getting more popular in this field. The following part of this chapter will review the influence of the support type on the oligonucleotide separation and extraction processes.

### 2.1. Silica

Fully porous silica particles were first used in OGN separation. Improved separation of OGNs can be achieved by applying adsorbents based on superficially porous particles. They are called core-shell or fused-core materials. The comprehensive studies on core-shell particles on RNA OGN separations were performed by Biba et al. [10]. They have compared fully porous (3 µm) and core-shell particles (1.9 µm solid core and 0.35 porous layer; total diameter 2.6 µm) with the same chemistry (C18). The core-shell particles ensure improved resolution and sharper peaks. They allow for better separation of impurities, e.g., higher resolution of compounds eluted before the main compound than fully porous support [10]. 

**Figure 3:**
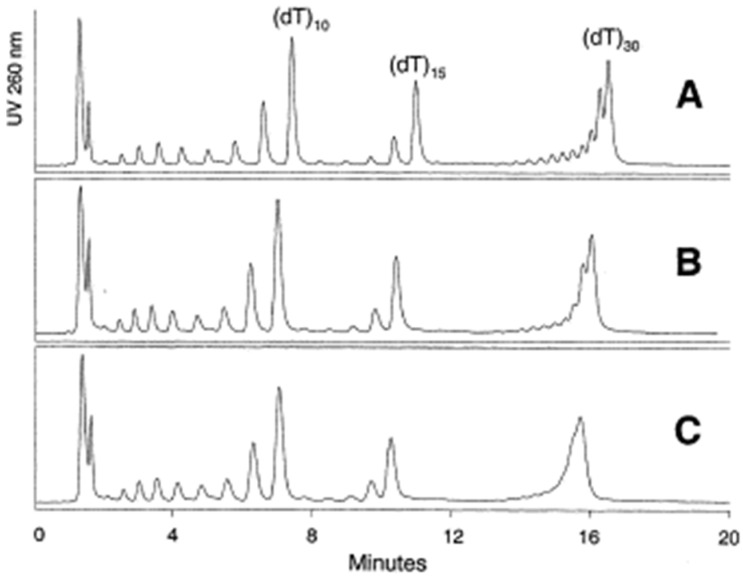
Impact of sorbent particle size on the separation of a 2–30 mer oligodeoxythymidine ladder. The separation was performed using an XTerra MS C_18_ 30 × 4.6 mm column packed with 2.5 μm (**A**), 3.5 μm (**B**), and 5 μm sorbent (**C**). The flow rate was 0.5 ml/min, and the column temperature was 50 °C. Mobile phase A: acetonitrile—0.1 *M* TEAA, pH 7 (5:95, *v*/*v*). Mobile phase B: acetonitrile—0.1 *M* TEAA, pH 7 (20:80, *v*/*v*). Gradient starts at 26.7% B (9% ACN); at 20 min it reaches 53.3% B (13% ACN). The gradient slope was 0.2% of acetonitrile per minute (0.4% per milliliter) (reprinted from Ref. [12], copyright 2021 with permission from Elsevier, license number 5177001267055).

Silica particle size is one of the basic properties of the stationary phase. The most popular in HPLC packed bed columns are particles ranging in size between 2.5–10 µm [10,11,12,13,14]. Particles with a diameter less than 2 µm exhibit higher efficiency and are used in OGN analysis in ultra-high-performance liquid chromatography. Gilar et al. [11,12] compared octadecyl stationary phases with fully porous 2.5, 3.5, and 5 µm particles for polydeoxythymidylic poly(dT) OGN separation of various length. All studied stationary phases allowed for the separation of shorter (sub-15 nucleotides) OGNs, but a visible broadening peak was observed for 3.5 and 5 µm particles. This effect can be explained by a slower mass transfer of biopolymers, while smaller particle sizes shorten the diffusion path and improve the separation (Figure 3) [11,12]. The OGN retention and separation tendencies differed for 10 µm adsorbents [13]. Such phenomena may be connected with changes in the primary conformations of OGNs during passing through particle-packed columns, especially those with small particle sizes [13]. The shortest analysis time and the best separation were obtained using 2.5 µm particles [11,12].

The consequence of improved efficiency was a shorter analysis time without losses in separation efficiency. After synthesis, small particles provided the best separation of closely related impurities in crude samples [10,11]. However, Cen et al. [13] reported some troubles with applying small particles for the quantitative analysis of OGNs due to low reproducibility [13]. Therefore, it should be underlined that for under 2 µm stationary phases, the narrow and tortuous channels restrict the retention of OGNs with secondary structures, such as hairpins [14]. 

Next to particle size, the most crucial parameter of silica adsorbents is their porosity. OGNs require the application of particles with large pores or non-porous particles. The negative effect of mass transfer can be reduced with core-shell particles [15]. Interestingly, Biba et al. showed that porous layer thickness influences OGN separation [10]. The narrower peaks were obtained for the column packed with particles of a smaller porous layer (0.35 µm) compared to those with the 0.5 µm layer [16]. On the other hand, Gilar and Bouvier [11] compared two columns packed with 5 µm adsorbents with 100 and 300 Å pores. No significant difference in separation performance was observed, proving that larger pores are not advantageous. Moreover, they suggested that the size of OGN fragments is not crucial for their diffusion throughout the pore of an adsorbent. Considering the size of the 30-mer poly(dT) fragment (~100 Å length and ~20 Å width) and pore size 100 Å, the conclusion is rather unpredicted [11].

### 2.2. Polymers

In OGN separation and extraction, polymers based on styrene-divinylbenzene (SDVB) [17], glycidyl methacrylate [17,18], glycidoxyethyl methacrylate [19,20,21], polymethacrylate [17], agarose [17], and regenerated cellulose [17] are used.

Deshmukh et al. [17] tested a few anion exchangers based on polymeric supports (SDVB, agarose, cellulose, and polymethacrylate) for OGN separation and large-scale purification. SDVB-based quaternary amine adsorbents appeared to be the best due to good chemical resistance in the pH range between 2 and 14 and the highest selectivity [17]. The addition of poly(vinyl alcohol) to the polymerization mixture resulted in a better separation of both OGNs groups. Similar tendencies were shown for polyvinyl alcohol with bonded diol groups [22] and hydroxymethyl methacrylate-based materials in HILIC [23]. An even higher resolution was obtained by modifying copolymers with C18 chains, which shielded the stationary phase from unwanted additional interactions with OGNs. Similar results were obtained from OGN separation, compared with polymeric adsorbents and nonporous or porous alkylated silica [23]. 

The methacrylate-based support minimizes hydrophobic interactions with OGNs but tends to hydrolyze under alkaline mobile phase conditions [19]. The glycidyl methacrylate stationary phase contains hydrophobic, ionic, and polar groups. Therefore, it can interact with OGNs by hydrophobic interactions, which were identified by Bunček et al. [18] as necessary during the analysis of OGNs under non-denaturing conditions. The combination of dehydration of adsorbents (exposure of the hydrophobic groups) and change of charge dipoles (more accessible hydrophobic interactions of bases) is responsible for the retention. Moreover, another type of support was tested for OGN separation, namely non-porous, hydrophilic polymer beads [24,25]. The binding capacity was lower than porous columns with the same ligand functionality. This stationary phase appeared to be very useful for OGN separation because peaks were very sharp with increasing resolution and reducing the analysis time [24,25].

### 2.3. Magnetic Nanoparticles

Magnetic nanoparticles (MNP) are becoming more popular in extracting different analytes from various samples [26,27,28,29,30,31,32]. The surfaces of MNPs are usually modified and functionalized with different molecules. Currently, the most commonly used material for coating the core of MNPs is silica. However, the polymer coatings of MNPs ensure a large surface area, stability, and biocompatibility. MNPs are also coated with silver or gold [26,32]. MNPs are applied in nucleic acid extraction, enrichment, and nucleic acid detection [32]. MNPs were also applied in OGN extraction, but the examples are very rare. In general, MNPs with strong anion exchangers (SAX) at the surface were investigated as a material for SPE of OGNs [33]. Moreover, a novel approach to OGN extraction requires the application of OGN-modified MNPs [34,35,36]. 

## 3. Application of Adsorbents with Different Modifications for Separation of OGNs

**Figure 4:**
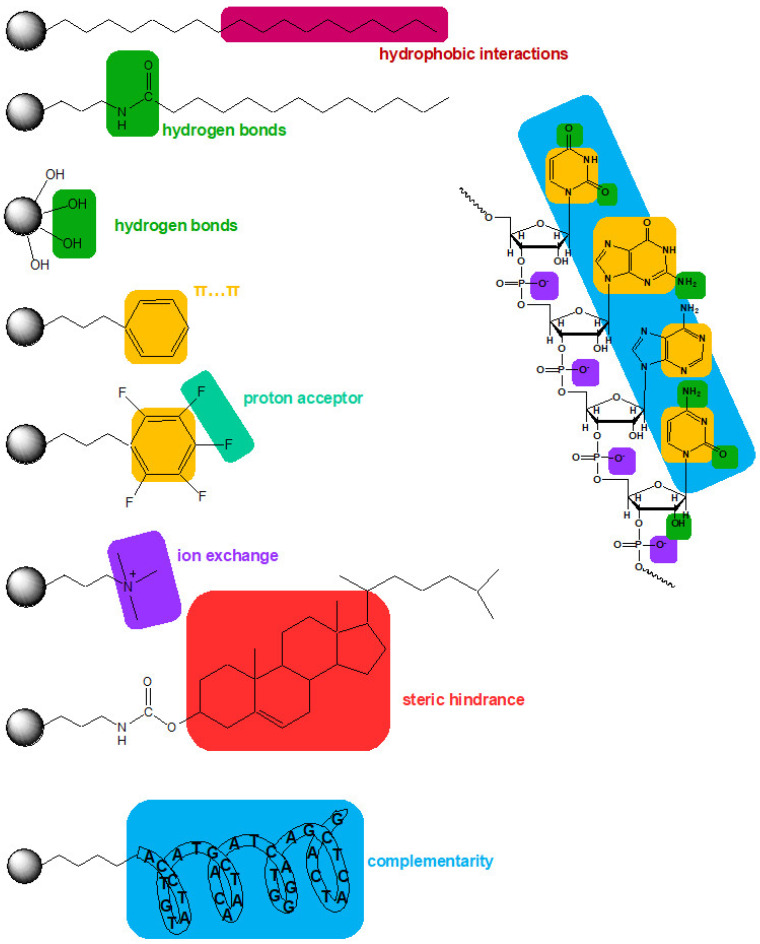
Schematic representation of possible interactions between the oligonucleotide and some stationary phases.

In addition to the support type and properties, the type of surface modifications also significantly impacts the separation and extraction of OGNs. The type of bonded ligand influences the retention mechanism and, at the same time, separation. Figure 4 presents schematic interactions between OGNs and the most typically used adsorbents.

### 3.1. RP Adsorbents

#### 3.1.1. Adsorbents with Alkyl Ligands

The C18 is the most popular adsorbent in chromatography and in OGN analysis applications. The retention of analytes at the C18 surface is based on interactions between hydrophobic parts of the molecule and the hydrophobic material surface [37]. Deprotected OGNs are polar and have multiple negatively charged moieties. For this reason, they have limited possibilities for interaction with non-polar materials. In order to solve this problem, a widely used strategy is the application of lipophilic ions as additives to the liquid phases used in the extraction or separation techniques. The ion pair model is based on interactions between hydrophilic OGNs, ion-pair reagents (with hydrophilic and hydrophobic regions), and hydrophobic adsorbents. Ion pair reagents (usually protonated alkylamine) reduce the charge of OGNs and, at the same time, form a layer with a positive charge on a solid phase surface [38].

Silica modified with C18 ligands is the most widely used adsorbent in the liquid chromatography separation of different OGN mixtures, such as unmodified DNA and RNA, phosphorothioate, 2′-fluoro, 2′-O-methyl modified OGNs, locked nucleic acid, and OGNs with triantennary N-acetyl galactosamine ligands [11,39,40,41,42,43,44,45,46,47,48,49,50,51,52,53]. One of the most straightforward attempts to separate OGN mixtures is single-stranded DNA/double-stranded DNA pairs [54,55]. According to Wysoczynski et al. [55], traditional approaches may fail for non-palindromic sequences, but IPC purification is proper. OGN retention depends on the sequence, so the sense/antisense pair separation is not complicated, similar to antisense and sense siRNA strands [51,54]. 

C18 modified silica is frequently used in IPC to separate shortmers, namely in vivo and in vitro metabolites [11,13,16,39,40,41,42,43,44,45,46,48]. Dai et al. [39] presented successful baseline separation of phosphorothioate ASOs and five metabolites shorter with one to five nucleotides from 3′-end. In separating OGNs shortened from one end, compound coelution is possible for a mixture of 3′-end and 5′-end metabolites [41,44]. Various stationary phases were tested to separate phosphorothioate OGNs and their metabolites (e.g., 5′ N-1, 3′ N-1, 5′ N-2, 5′ N-3), but the C18 column provided the best separation efficiency and highest results reproducibility [41,44]. Despite optimizing separation conditions, coelution of isobaric 3′ and 5′ metabolites was noticed [41,44]. Chromatographic separation of such OGNs is still challenging. Despite this fact, C18 is useful for OGN separations and metabolite profiling in biological samples such as plasma, urine, tissue (liver, kidney) from rats, mice, and humans [13,39,40,41,42,44,45,49,50]. It is also used in vitro OGN metabolism studies in different matrices: fresh human blood, human serum and liver microsomes, mouse tissue homogenates, and rats hepatocytes [43,45,46,47].

Some scientists performed OGN retention studies with the same base composition but with different sequences (sequence isomers) on C18 stationary phases [56,57,58]. Consequently, they reported differences in the retention: the change of nucleobases near the 3′ or 5′ end contributed to the retention greater than changes in the middle positions of the sequence [58]. Moreover, the selectivity factor was higher for OGN pairs differing in the type of terminal nucleotides compared to pairs with various internal positions of nucleobases. The access of 3′- or 5′-end of OGN to the stationary phase surface is greater than a large hairpin loop [56,57]. The retention of these biomolecules depends not only on the size of the molecule but also on base composition and sequence [57].

Furthermore, the C18 stationary phase is the most suitable to separate OGNs differing in one base position in the sequence when the IPC is used [56]. These results prove that the C18 column may be successfully used for sequence isomers separation, e.g., multiple impurities in crude samples after synthesis. Nikcevic et al. [59] tested a group of chromatographic columns with vendor specific C18 stationary phases to separate and profile low-level phosphorothioate OGN synthesis impurities. The developed method allows for the separation of many impurities: shortmers, N-mer with terminal phosphorothioate, depurination product n-mer, and some adducts [59].

Another challenging task in OGN separation is the resolution of phosphorothioate OGN diastereomers with potentially different pharmacological and physicochemical characteristics [60]. Several investigations showed the usefulness of C18 stationary phases to separate OGN diastereomers [60]. Up to tetramer OGNs were successfully separated, but it was shown that the elution order of RS and SR diastereoisomers cannot be deduced from the sequence and indicates that there are no straightforward analogies even for simple diastereoisomeric mixtures. However, ion pair reagent type, the OGN sequence, and the number and position of sulfur substitutions influence the retention behavior and selectivity [60]. The separation of all diastereoisomers may be achieved by a small degree of substitution of O by S in the OGN backbone. For the fully substituted 5-mer poly(dT), 16 diastereomers are expected, but only eleven were separated [60].

**Figure 5:**
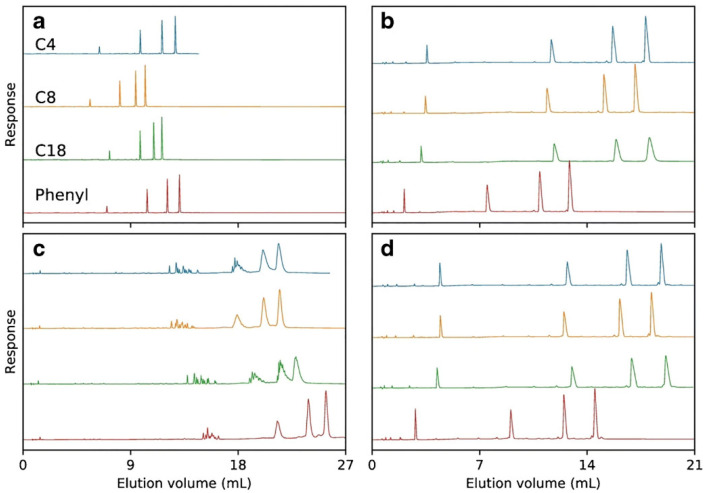
A mixture of T5, T10, T15, and T20, separated on Kromasil C4, C8, C18, and phenyl columns using either triethylammonium acetate (TEtAA) or tributylammonium acetate (TBuAA) as an ion-pairing reagent. Plot (**a**) shows chromatograms using native oligonucleotides eluted in 50 mM TEtAA. In plot (**b**), the same sample is separated using 5 mM TBuAA. Plots (**c**,**d**) show the elution profiles of a PSmodified oligonucleotide, under identical conditions as in (**a**,**b**), respectively (reprinted from Ref. [62], Open Access article distributed under the terms of the Creative Commons Attribution 4.0 International License).

Despite the wide application of C18 adsorbent, other alkyl modified silica materials were also applied for OGN analysis [11,53,56,61], as it is well known that alkyl chain length influences retention. It was observed that lower hydrophobicity of shorter alkyl chains bonded to adsorbent surface effects in lower strength of interaction between OGNs and the stationary phase [53,56]. Consequently, the utilization of the octyl stationary phase is not suitable for separating OGNs mixtures containing sequence isomers and, in some cases, ASO metabolites [53,56]. On the other hand, the diastereomeric selectivity for phosphorothioate OGNs was the highest for C18 columns and was reduced with increasing OGN length [62] (Figure 5).

#### 3.1.2. Adsorbents with Aromatic Groups

The next group of stationary phases tested in OGN separation are materials with bonded aromatic ligands. Two types are dominant: aryl and pentafluorophenyl adsorbents [10,53,56,63,64]. The aryl groups can interact with OGNs through π–π interactions, which take part in the base-stacking effect [43,53,56,63]. Introducing fluorine atoms to aryl rings (pentafluorophenyl) changes the selectivity compared to the C18 and aryl stationary phases. The electronegative fluorine atoms cause preferential interactions with analyte donating protons. The aryl stationary phase has an electron-rich aromatic ring, which increases OGN retention (Figure 4) [43,56,63]. Higher retention factors are usually determined for the aryl stationary phase, but this is not a general rule [10,43,56]. Similar retention for OGNs at octadecyl and aryl stationary phases was observed in some cases. The utilization of the pentafluorophenyl adsorbent provides lower OGN retention than alkyl and arylcolumns [53,56]. Nonetheless, the baseline separation of OGN sequence isomers for phenyl and pentafluorophenyl stationary phases was shown [56], ASO mixture with different types of modifications for pentafluorophenyl [53], ASOs for aryl [56]. On the other hand, pentafluorophenyl have failed during an attempt of phosphorothioate OGNs and their metabolites separation, contrary to the aryl stationary phase [43,62]. OGNs were also studied with similar aryl groups (pentafluorophenyl and phenyl) bonded to long C18 chains RP HPLC [63]. OGN retention was the lowest for C18 with pentafluorophenyl. Greater retention observed for C18 with terminal phenyl allowed for successful separation of various OGN mixtures: modified and unmodified of different lengths or sequences [62,63] (Figure 5).

The effect of the spacer between the aryl group and silica surface was tested for the phenoxy-propyl, phenyl-propyl, and phenyl-hexyl stationary phases [64]. The lowest retention factors were obtained for a spacer with an oxygen atom, while the greatest were for phenyl-hexyl [64]. These differences are related to the effect of an even or odd number of carbon atoms in the spacer. The orientation of the phenyl ring is parallel to the alkyl spacer chain when the spacer chain contains an even number of carbon atoms promoting π–π interactions. The opposite situation appears when there is an odd number of carbon atoms [64]. Such changes in the conformation cause various retention effects observed for OGNs with different lengths (retained mainly by π–π interactions) and sequence (retained mainly by electrostatic and hydrophobic interactions) in IPC [64]. The best separation selectivity of OGNs differing with the length and sequence was observed for the phenoxy-propyl stationary phase. 

Aromatic groups are also present in the structure of polymer based SDVB adsorbents. Xiong et al. [65] compared this stationary phase with C18 in the separation of isomeric adducts derived from the covalent binding of (±)-anti-7r,8t-dihydroxy-9t,10-epoxy-7,8,9,10-tetrahydrobenzo[a]pyrene to OGNs. Poor chromatographic performance of these analytes was observed on the C18, while for SDVB the peaks of the OGN adducts were partially resolved at elevated or room temperature. The π–π stacking interactions between the aromatic rings of the SDVB and the adducted base may partially account for the improved retention and separation of the analytes [65].

Therefore, the SDVB stationary phase was successfully used for unmodified and modified OGNs (phosphorothioate, phosphorothioamidate, 2′-fluoro, 2′-O-methyl, 2′-O-methoxyethyl, different 5′-end, and 3′-end modifications, and cancerogenic adducts) analysis [35,65,66,67,68,69,70]. To conclude, polymeric adsorbents with aryl groups offer similar possibilities as silica supports, but their resistance to high pH is much higher. This shows its advantage over C18.

#### 3.1.3. Adsorbents with a Cholesterol Molecule

The cholesterol bonded phase was also tested for OGN separation in IPC [57,71]. OGN retention increases for the cholesterol stationary phase in comparison with C18. The presence of double bonds in the cholesterol structure causes additional interactions with OGNs. Moreover, OGNs can interact with unreacted aminopropyl ligands. Thus, higher retention of these polar molecules can affect different interactions with multiple functional groups of complex ligands (Figure 4) [57]. The cholesterol stationary phase was used to separate OGN sequential isomers and OGNs of different lengths [57,71]. The access of OGNs to the surface of this stationary phase is different. Therefore, the selectivity was not influenced by changes in the terminal or internal positions of nucleotides in OGNs [57]. This stationary phase exhibited another interesting property, connected with the impact of ion pair reagents on the OGN retention. The retention usually increases with amine concentration in the mobile phase in IPC. The opposite effect was observed for the cholesterol stationary phase and triethylamine, for which OGN retention was greater when low amine content in the mobile phase was used [71]. This effect may be related to micelle formation [72]. Consequently, this adsorbent allowed both: the application of a low triethylamine concentration and obtaining high k values [71]. However, based on our experience, OGN resolution for this stationary phase was limited to the three, four-component mixture.

#### 3.1.4. Adsorbents with Polar Groups Incorporated in Non-Polar Ligands

The alkylamide stationary phase was also applied to OGN studies in RP HPLC and IPC [57,73]. Compared to C18, the retention of OGNs was lower [56,63]. The polar heteroatoms implemented in alkyl chains (e.g., in the amide group) increase the polarity of the stationary phase and, at the same time, reduced the retention of OGNs (Figure 4) [53,57,64]. It was also shown that OGN retention increases with the decrease of the mobile phase pH in RP mode, indicating the critical role of surface charge change [73]. The advantage of the alkylamide stationary phase is that the application of RP HPLC allowed for the successful separation of sequence isomers at acidic pH containing a high concentration of buffer. Contrastingly, the time needed for separation was long, and peak asymmetry was significant due to the amide and aminopropyl groups’ presence [73]. Greater OGN peak symmetry was obtained for a similar stationary phase when UHPLC was used in ion pair mode [63,74]. It was successfully used to separate phosphorothioate OGNs of different lengths with greater resolution, selectivity, and shorter times than C18 and phenyl stationary phases [63,74]. On the other hand, applying this stationary phase to separate sequence isomers has failed [53]. 

To sum up the role of RP adsorbents in OGN separations, it has to be pointed out that alkyl stationary phases are currently the best choice for OGN analysis, especially C18. It provides very good resolution, selectivity, and reproducibility. Similar results may be obtained for aryl-based stationary phases used in IPC and RP HPLC, but phenyl-based ligands without fluorine atoms should be applied. On the other hand, a limited number of applications for this type of stationary phase are described in the literature. Therefore, there is a need for further investigations, e.g., the separation of more complex metabolite sets, or similar. 

### 3.2. Adsorbents with Anion Exchange Groups

The ion-exchange mode is mainly used in the OGN separation. Their retention is based on the electrostatic interaction between the negatively charged analyte and positively charged adsorbent surface, taking advantage of the charge on the phosphate linkage (Figure 4) [75,76]. These adsorbents can be divided into two groups: strong (with permanent charge) and weak anion-exchangers (with secondary or tertiary amine at the surface) [76].

**Figure 6:**
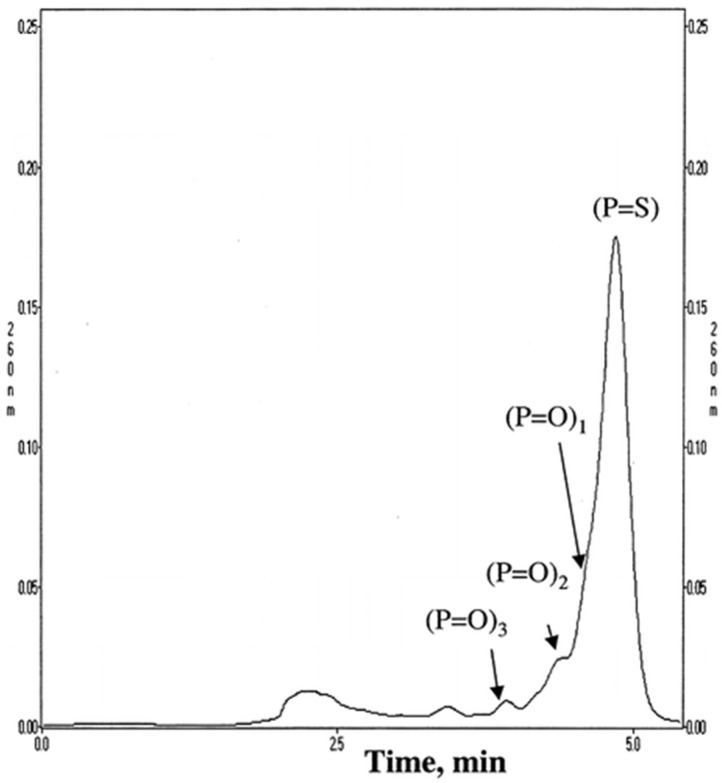
Representative runs without optimized conditions. Column:  Resource Q 1 mL (6.5 mm i.d. × 30 mm length). Buffer A:  20 mM NaOH. Buffer B:  20 mM NaOH/2.5 M NaCl, flow rate 5 mL/min, gradient 0−100% B in 20 CV, loading 50 μg (50 μl of 1 mg/mL ISIS 2302) (Reprinted from Ref. [62] Open Access article).

Adsorbents with quaternary ammonium ligands are frequently used in chromatographic OGN analysis by IEC [17,19,20,21,75,76,77,78,79,80,81]. It is difficult to achieve complete length-based resolution for most anion exchangers, e.g., 19-mer phosphorothioate is usually not entirely resolved from the 20-mer phosphorothioate, contrary to phosphodiesters of similar lengths. On the other hand, the application of SAX is sufficient to get a suitable OGN purification. Deshmukh et al. [17] optimized conditions for chromatographic purification of crude phosphorothioate OGNs using a wide variety of SAX stationary phases: quaternary amine ligands including diethylaminoethyl and trimethylaminoethyl. The stationary phases with the quaternary ammonium group allowed for the complete separation of phosphorothioate ASO impurities, phosphorothioate products, and their unmodified analogues (Figure 6). Moreover, SAX was successfully applied to separate unmodified, single-stranded OGNs, such as the main compound from tritylated precursor; OGNs with identical lengths but different sequences or base composition; OGNs with different lengths [19]. Polystyrene-based quaternary amine SAX may also be used to purify ASO and length-based separation (e.g., phosphorodiamidate morpholino OGNs) during the in vitro bioavailability pharmacokinetic studies [17,77,78]. It must be stated that although the complete separation of OGNs and ASOs is generally obtained, the time needed for a single analysis was very long (up to 30–50 min for a three to ten component mixture) [78]. Interaction of OGNs with strong anion exchange groups at the stationary phase surface is strong. Therefore, retention times are usually long. They may be reduced by changing conditions shown by Yang et. al. (baseline separation of OGN phosphorodithioates and diastereoisomeric impurities for 20 min; conditions: buffer A: 25 mM Tris–HCl, 1 mM EDTA, pH 8; buffer B: 25 mM Tris–HCl, 1 mM EDTA, 1 M NaCl, pH 8; linear gradient: 0.0–100.0% B in 80 min; flow rate: 2 mL/min) [79]. 

Since porous anion-exchangers provide sufficient capacity for lab-scale purifications, they exhibit very low throughput, low resolution, or lengthy analysis times (due to slow mass transport) [17,19,21]. The superficially porous IEC adsorbents with quaternary ammonium groups were applied for OGNs analysis to overcome these limitations. Pellicular anion-exchangers have been used for the separation of DNA containing intrastrand cross-links [82] and phosphorothioate OGN diastereoisomers [83] isobaric RNA linkage isomers, as well as phosphorothioate diastereoisomers of DNA and RNA [80]. These stationary phases promote convective mass transfer. Consequently, a substantial increase in OGN capacity (retention and peak shape) over other pellicular and porous materials is observed [80]. It is important to notice that there is a significant reduction of time needed for the separation compared to porous stationary phases. This type of material probably has the most significant future for OGN analysis by IEC. 

Various weak anion exchange (WAX) can be used to separate OGNs, but the most common one is diethylaminoethyl adsorbent [18,84]. The pH is a critical parameter for the retention of OGNs on the surface of this adsorbent. The diethylaminoethyl has a maximum surface charge at pH~4. With increasing pH, the charge decrease, and at pH~9, it is approximately 50% of the maximum value [18]. At the same time, the formation of secondary structures of OGNs at pH 8–9 is the lowest [18]. Consequently, the separation of OGNs strongly depends on adsorbents and OGNs secondary structure [18,24,25]. 

The diethylaminoethyl was used to separate OGNs differing with the type and position of one base in the sequence [18,24,25]. It was concluded that OGNs interact with the DEAE stationary phase mainly at their 5′ and 3′ ends and interaction of the interior bases with the stationary phase is relatively small [18]. WAX can be useful for separating all potential N-1 deletion sequences and a few positional isomers of various homooligonucleotides [18,24]. However, it must be highlighted that WAX may be used for OGN separation based on the chain length at the mobile phase of pH 8.5–9.5. On the other hand, OGN separation according to the base composition is possible by using an eluent of high pH of around 10.5 [18,24]. The peaks were tailing when mobile phase pH was below 7.5. These effects are a consequence of inter alia deprotonation of guanine and thymine in the pH range of 9–10, and protonation of cytosine, adenine, and guanine at pH lower than 4.5. 

Generally, applying ion-exchange materials as stationary phases in liquid chromatography allows employing elevated temperature or pH to control OGNs separation selectivity by producing fully or partially denaturing conditions [75]. Moreover, it should be highlighted that utilization of SAX as stationary phases provides good separation, although the time of analysis is long. In some cases, the OGN separation selectivity is lower for WAX, but the analysis time is shorter than SAX. Anion exchange using polymeric pellicular resin technology is a robust and stable methodology for N-1 failure sequence analysis for both unmodified and modified ASO impurities; for the resolution of phosphorothioate diastereoisomers as well as for antisense and sense strand analyses in double-stranded RNA. Generally, the resolution of OGN mixtures is comparable for alkyl and ion-exchange stationary phases. However, ion exchange materials can be successfully used in cases where there is no need for a mass spectrometer.

### 3.3. Polar ADSORBENTS

#### 3.3.1. Adsorbents with Hydroxyl Ligands

The adsorption of DNA on unmodified silica is possible in chaotropic salts and buffers, which ensure deprotonating of silanol groups and high ionic strength. DNA can be eluted with salts of low concentration [85]. Nowadays, unmodified silica is also used in HILIC to analyze OGNs [22,23,25,86,87,88,89]. Li et al. [86] presented base-line separation of a 27 poly(dT), poly(dA) and poly(dC) homooligonucleotides mixture during 100 min [86]. The most critical chromatographic parameter influencing the retention appeared to be the pH of the mobile phase: OGNs retention increased with decreasing pH. It was associated with increased protonation of the silanol groups and ionization of the phosphoryl groups in the OGN structure, which reduced the repulsion between both species [86]. Studzińska et al. [90,91] also attempted to apply silica as a stationary phase for the HILIC analysis of OGNs. Unfortunately, these compounds were irreversibly retained at the surface of the stationary phase, or the shape of the peak was very asymmetrical, causing the lack of separation or even OGNs coelution [90,91]. A different attempt was presented by Gong [87] as instead of mobile phases typically used for the OGNs separation in HILIC mode, he applied the mobile phase commonly used in IPC [87].

The retention mechanism of OGNs at bare silica surfaces is based on hydrogen bonding and ion exchange (when the pH of the mobile phase is changed) [85,86,90,92]. However, this mechanism changes when ion-pair reagents are used as mobile phase additives [87]. The protonated amine ions form ion pairs with negatively charged phosphate groups of OGNs and undergo hydrophilic interaction with the water-rich layer at the surface of the stationary phase [87].

Researchers investigated other adsorbents with hydroxyl groups useful for separating OGNs [22,87]. For example, by applying diol modified silica, they reported poor column stability, poor retention time reproducibility, and peak symmetry [88,90,91,93]. These effects were also observed by Easter et al. [88], who applied silica-based columns with crossed diol functional groups for homooligonucleotides separation. Here, the low peak area reproducibility was noticed. Consequently, this stationary phase required reconditioning periods of at least 30 minutes, and its application was limited [88]. For these reasons, Lobue et al. [22] tested the stationary phase with diol groups at the polymer-based particles. The authors performed a few successful OGN mixture separations: poly(dT) DNA OGNs with different lengths; synthetic RNA OGNs with different lengths and common nucleosides modifications; methylated OGNs and their unmethylated counterparts; sequence isomers; phosphorothioate OGN from closely related failure sequences [22]. The range of applicability of this stationary phase was extensive. Worth mentioning is that in the case of all tested mixtures, the time needed for the separation did not exceed 30 minutes for 20 and 27 compounds. Moreover, this was done with HILIC mobile phases without ion-pair reagents [22,87].

#### 3.3.2. Amide Adsorbents

The amide stationary phase was used to separate homooligonucleotides (dT) and phosphorothioate ASO [88,89,90,94]. The amide group allowed for an increase in the role of hydrogen bonding in the OGN retention mechanism. Moreover, a partition mechanism was identified when this stationary phase was used in HILIC [88]. This stationary phase exhibited high resolution of OGN and ASO mixtures under gradient conditions without mobile phase modifiers in less than 25 minutes [88]. Moreover, it was successfully used for the separation of phosphorothioate ASO mixtures (differing by the sequence and length) for a maximum of 15 minutes (for five-component mixtures) [90]. The application of amide column in HILIC provides reproducible retention of symmetrical OGN peaks with low tailing [90,94]. This is extremely important for the routine analysis of OGNs, and it is difficult to achieve with a diol or silica-based stationary phase [88,90,93]. However, despite the advantages of amide adsorbent use, column conditioning is still required for HILIC (regeneration time of 20 minutes) [88,89,90]. Lately, Goyon and Zhang [81] used the amide stationary phase in the second dimension of 2D-LC to separate and desalting of preseparated phosphorothioate OGNs. The mobile phase’s pH was critical because the ASO did not elute at acidic pH, while neutral and basic conditions allowed ASO separation [81].

#### 3.3.3. Zwitterionic Adsorbents

**Figure 7:**
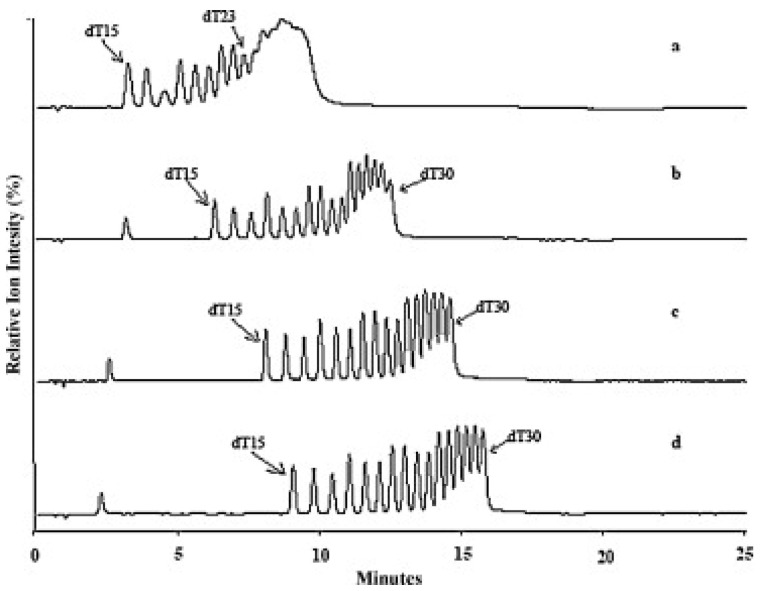
Separation of oligonucleotides dT15-30. PEEK ZIC^®^ – HILIC, 100 mm × 2.1 mm, 3.5 μm column. Mobile phase A: Milli-Q H_2_O; B: acetonitrile; C: 100 mM ammonium acetate, pH 5.8. Gradient from 70% to 60% B in 15 min, with constant C 5% (**a**), 10% (**b**), 15% (**c**) and 20% (**d**), flow rate, 0.6 mL/min but only 0.2 mL/min split to MS, temperature, 50 °C; 10 picomole each injected (reprinted from Ref. [93], copyright 2021 with permission from Elsevier, license number 5177000944265).

Another group of adsorbents used in separating OGNs in HILIC is zwitterionic. Some investigations suggest that this stationary phase is the best for separating OGNs [93,95]. Gong and McCullagh [93] compared them with hydroxyl stationary phases. Zwitterionic column ensured high signal sensitivity and allowed for the separation of a mixture of poly(dT) with 15–30 nucleotide length, few heterogeneous OGNs, as well as N-1 shortmers and methylated analogous (Figure 7) [93]. Analogical analytical studies were performed by Studzińska et al. [91]. They have compared the zwitterionic (sulfobetaine) adsorbent, polyhydroxy, aminopropyl, and bare silica in HILIC. However, satisfactory results were obtained in just the sulfobetaine stationary phase. It was applied to separate 2′-O-(2-methoxyethyl) modified OGN and its two synthetic metabolites [91].

Polar adsorbents may provide high resolution when a proper stationary phase (amide or zwitterionic) is used together with a mobile phase of suitable pH. The critical point is the peak symmetry, which may be very high. Their use in oligonucleotide analysis may have an advantage if HILIC and mass spectrometry are needed. Regarding the application of silica gel and stationary phases with diol groups, their utilization will not provide good results in terms of resolution or reproducibility. Therefore, they should not be used.

### 3.4. OGNs-Modified Adsorbents

Although it is not common yet, affinity chromatography separation/purification is an efficient strategy for OGN analysis [96]. The binding between the OGN and the affinity ligand is selective, reversible, and capable of isolating and identifying target molecules complementary to a specific OGN sequence bonded to a silica surface (Figure 4). Affinity chromatography was applied for selective fractionation of nucleic acids and OGNs, their purification, separation, and studying interactions of DNA with other molecules [97,98]. However, this technique was infrequently used for separation based on the hybridization effect involving helix formation between specific DNA sequences and an immobilized OGN [98,99]. Usually, OGNs coupled with solid support form a complementary double strand with the studied OGN, while other molecules present in the sample (proteins, nucleotides, plasmid DNA, double-strand RNA, and enzymes) are not retained at such stationary phase surfaces. The non-complementary sequences are eluted in the first fractions of eluate, while all sequences complementary to the attached ones are retained at the stationary phase surface [99]. This separation appeared to be very selective and efficient. Similar effects were observed for plasmid DNA purification, proving affinity chromatography may be applied to isolate target OGNs from complex biological samples [98]. Moreover, these stationary phases may be applied for the analysis of short nucleic acids fragments, as it was presumed that short and modified OGNs do not form stable complementary complexes with the affinity template under hybridization conditions. The affinity chromatography may be used to separate various OGNs of similar length but differing in complementarity degree (various number of base mismatches) and the separation of octathymidylate containing three nonionic methylphosphonate backbones and forming eight diastereoisomers [99]. Furthermore, the OGN-based stationary phase was applied for ASO hybridization studies [99].

The DNA-based affinity columns are usually equilibrated with a hybridization buffer. The elution of a target biomolecule bound to the affinity ligand is achieved by changes in the mobile phase composition (type of salt and ionic strength, pH, temperature). Usually, elution is performed with water, urea, or heat denaturing [99]. Generally, the temperature is the main factor influencing the selective separation of OGNs in this mode of chromatography.

The silica support modified with OGNs was successfully used to extract these compounds [100]. Selectivity studies revealed that no noncomplementary OGNs are retained at the surface, and the adsorption percentage increases with the decreasing number of base mismatches. Silica-based adsorbents with immobilized OGNs were used for the selective extraction of OGN and its metabolites from serum samples with recoveries in the range of 65–73% for both unmodified compounds and ASOs [100]. These values were lower than MNPs modified with OGNs, probably due to the nonspecific adsorption of tested compounds at silica support. 

According to the literature, affinity chromatography with OGN-based stationary phases is a powerful technique for the specific base recognition of polynucleotides. On the other hand, it has two disadvantages: cost (each column is tailor-made) and column lifetime (silica-based affinity stationary phases have a limited lifetime due to mobile phases applied). 

## 4. Application of Adsorbents with Different Modifications for Extraction of OGNs

C18 adsorbent can be used in SPE for OGN extraction, mainly for phosphorothioate and single and double-stranded DNA. It was used to extract these analytes from mammals’ plasma (including humans) [16,55,61,66,101]. The application of C18 for the extraction of ASOs (e.g., phosphorothioate) provided recoveries approximately 80% [61,66,101]. However, the application of modified silica for the extraction can cause analyte losses. 

The introduction of a polar group into the adsorbent structure worked well for extraction purposes. Oasis HLB is a mixed-mode copolymer of divinylbenzene (nonpolar part) and N-vinylpyrrolidone [102]. It is often used in the SPE extraction of phosphorothioate, 2′-fluoro or 2′-O-methyl OGNs and their metabolites from different biological samples (human, monkey, mouse, and rat plasma, rat urine, mouse liver, and kidney homogenate), as well as samples from in vitro incubation with enzymes (3′ exonucleases), human liver microsomes, and primary rat hepatocytes [11,13,39,40,41,42,43,44,45,46,47,48,49,66,68,101]. Typically, the OGN recovery values for Oasis HLB columns ranged between 60–80% [11,13,39,40,41,42,43,44,45,46,47,48,49,66,101]. Moreover, purification of crude, unmodified DNA OGNs with Oasis HLB gave 90–95% purity with a recovery of the target compound of 60–96% [11].

SAX with quaternary ammonium ligands was also demonstrated as an effective material in extracting phosphorothioate OGNs from human plasma with high extraction recovery (>90%) and good separation of OGNs from proteins. However, the extracts must be desalted because highly concentrated salts are for elution. The most significant disadvantage of ion exchange SPE is utilizing over 1 molar concentration of inorganic salts for elution. These salts are also used to elute OGNs from DEAE adsorbents, so the desalting step is also required. Ye and Beverly [33] made a similar attempt, but they used volatile salts (triethylammonium bicarbonate, ammonium bicarbonate, and ammonium chloride) for the elution of siRNA. Consequently, speeding up the overall time of analysis because the desalting step was omitted. For this purpose, the quaternary aminoethyl functional groups bonded to the surface of magnetic nanoparticles were used. The recovery of OGNs depends on the hydrophobicity of OGNs, their length, and modification type, but usually it equals 80% [33].

The most popular adsorbent used for routine OGN extraction is the Clarity OTX^®^. It is a polymer-based (surface-modified PSDVB) mixed-mode WAX adsorbent, for which the information about the exact structure of the functional groups bonded to the support is protected by the manufacturer. It was used to extract DNA, RNA, phosphorothioate, thiophosphoramidate, 2′-O-methyl, 2′-fluoro, 2′-O-(2-methoxyethyl), 2′-4′ locked, lipid or N-acetylgalactosamine modified OGNs from human and other mammals’ plasma, urine, liver, kidney, and brain homogenate samples [35,36,51,52,54,69,70,103]. The sample loading is performed after lowering the pH to 5.5, while the elution is performed with a mixture of buffer (pH equal 9.5) and an organic solvent. This adsorbent allows for the avoidance of high concentrations of inorganic salts for elution [103]. In general, the extraction recovery is in the range 60–100% [35,36,51,52,54,69,70,103]. They were usually higher for plasma samples than for tissues. High (usually >80%) recoveries for tissue homogenates were obtained for OGNs phosphodiester and phosphorothioate linkages and 2′-O-methyl or 2′-fluoro modifications. Lower (60–30%) recoveries were obtained for phosphorothioamidate and phosphorothioate OGNs with 2′-O-(2-methoxyethyl) or N-acetylgalactosamine modifications. The lowest recovery values (<20%) were obtained for OGNs with nonpolar 5′-end modifications, like tocopherol or cholesterol [35]. 

Another adsorbent is Oasis WAX, a copolymer of divinylbenzene and N-vinylpyrrolidone modified with piperazine group [104]. Similar to Clarity OTS^®^ adsorbent, the application of Oasis WAX^®^ does not require the utilization of a high salt concentration for OGNs elution, e.g., 2% ammonium hydroxide in (3:7 acetonitrile: water) [50,94]. SPE using this adsorbent was presented as an effective tool for extraction of unmodified and phosphorothioate OGNs from human plasma samples with recoveries ranging between 50–68% [50,94]. These values may be increased. However, a higher concentration of inorganic salts would have to be used.

Silica is rarely applied to OGN extraction due to a relatively low recovery between 55–60% [92]. The hydrophilic interaction mode was applied, involving sample dilution and loading with acetonitrile or acetone and elution using water. The procedure is straightforward, and no salts of high concentration are needed. It may be a promising alternative to popular techniques used at the moment [92].

The hybridization-based extraction of OGNs is based on the annealing of sense and antisense strands. Currently, the most commonly used are MNPs modified by OGNs. The antisense probe (strand that contains 12–15 complementary bases) usually includes a biotin label for affinity capture of the sense–antisense complex from the sample [36,105]. This allows for the isolation a specific complementary sequence from complex matrices. This approach has been applied to mRNA, miRNA, and ASO extraction [33,35,105,106]. 

Dillen et al. [36] used hybridization-based extraction for the isolation of novel ASO-imetelstat (13-mer OGN thiophosphoramidate with a covalently linked palmitoyl lipid moiety at the 5-end) from preclinical (rat plasma) and clinical (human plasma) samples. They succeeded with high recovery, and results were comparable to SPE extraction using a weak anion exchanger. Dillen et al. [36], as well as Kim et al. [107], suggested that SPE with weak anion exchangers should be considered as a more standard approach, mainly when multiple OGNs are extracted (a native compound with metabolites). Hybridization extraction has more significant potential, mainly due to its selectivity (which increases recovery), but critical reagents per analyte are needed (tailor-made OGN sequences which are complementary to strand bonded to the support) [36,107]. Application of biotinylated capture strand coated magnetic beads (complementary with eluforsen) to extract eluforsen, and its metabolites showed that only N-1, N-2, and N-3 metabolites (3’ end shortmers) were detected. More 3’ end shortmers (from N-1 to N-8) were observed after the SPE method [107]. These results indicate that hybridization-based extraction may be characterized by a specific cutoff fragment length for which the capture strand loses affinity and selectivity. So far, this is the main drawback of such an extraction attempt. However, if it is not necessary to recover metabolites, this is undoubtedly a powerful approach with very high OGN specificity and good recovery (80–95%) [29,105,107,108]. Moreover, many other advantages of this approach were listed, namely: improved sensitivity, selectivity, and even quantitation capability for truncated metabolites; better tissue sample compatibility simpler method development; tighter precision and accuracy; lower matrix effects [107,108].

The silica support modified with OGNs was successfully used to extract these compounds [100]. Selectivity studies revealed that no non-complementary OGNs are retained at the surface, and the adsorption percentage increases with the decreasing number of base mismatches. Silica-based adsorbents with immobilized OGNs were used for the selective extraction of OGN and its metabolites from serum samples with recoveries in the range of 65–73% for both unmodified compounds and ASOs [100]. These values were lower than MNPs modified with OGNs, probably due to nonspecific adsorption of tested compounds at silica support. 

To summarize, it should be emphasized that extraction based on electrostatic interactions and hybridization seems to have the highest potency for isolation of OGNs. The use of non-polar adsorbents, or those with alkyl ligands, necessitates the use of reagents for ion pair formation. This, in turn, is associated with an increased produce time and dependence of recovery on the ion pair formation efficiency. The application of SAX allows to obtain high recovery, but the use of high salts concentration leads to the desalting necessity. OGN extraction using WAX provides high recovery and LC-MS friendly solvents, and this SPE material is currently one of the best choices [75], especially for OGN extraction, contrary to polar adsorbents application. They have several disadvantages, e.g., silica gel allows to obtain about 50% of the recovery, but it may cause irreversible OGN adsorption when organic salts are used as mobile phases. Other materials of great potential for OGN extraction are OGN-based materials used in hybridization extraction. They provide excellent selectivity. However, the disadvantage here is the need for tailor-made OGNs. Moreover, the recovery is very high for complementary OGNs, while it is decreasing for shorter (metabolites) sequences.

## 5. Future Perspectives

A wide variety of very different materials have been used to date in the separation and isolation of OGNs. Their advantages and disadvantages are presented in the above review. An overall comparison was also presented in Table 1. Considering the supports, the most analytical problems are observed for silica ones, while magnetic nanoparticles are the best for OGN extraction. With chromatography, the best results were obtained for polymeric or surface-porous supports. We believe that both types of support are optimal, and there is little to be done in terms of OGN analytics. However, considering the groups bonded to the surface of these supports, we believe that intensive development is still necessary for this area. This is especially important for liquid chromatography and stationary phases. Hydrophobic adsorbents are currently the most commonly used. They provide high resolution and a short analysis time for ultra-high-performance liquid chromatography. However, it is necessary to use amines in the mobile phase, which may reduce sensitivity. On the other hand, carefully optimized concentrations of ion pairs increase electrospray sensitivity. Mobile phases lowering the sensitivity of the determination are also characteristic of ion exchangers, very polar adsorbents, or stationary phases with bound oligonucleotide molecules. Development in this area should focus on the synthesis and application of stationary phases to retain OGNs on their surface and allow separation without mobile phase additive usage. It would be possible to use mobile phases consisting of water and organic solvent with the addition of a volatile acid. This is a challenging task due to the properties of OGNs.

Nevertheless, efforts in this area should be significant among researchers. The adsorbents currently used to extract OGNs provide satisfactory results. In situations where high selectivity of a given oligonucleotide is required, MNPs modified with OGNs molecules should be used. Meanwhile, if recovery of all OGNs from the sample is essential, then high values of this parameter are provided by weak ion exchangers and adsorbents with hydrophobic or aromatic groups.

## Figures and Tables

**Table 1:** The rough outline of functional groups impact on selected LC and SPE parameters for OGNs analysis.

Functional Groups at the Surface of the Support	Parameter					References
	LC			SPE	Types of Analyzed OGNs	
	Resolution	Selectivity	Time	Recovery		
Alkyl chains	high	high	short	very high	homoligonucleotides, shortmers, longmers, metabolites, sequence isomers, depurination products, ASO, phosphorothioate OGNs diastereoisomers	[10,11,13,16,59,36,39,40,41,42,43,44,45,46,47,48,49,50,51,52,53,54,55,56,57,60,61,62,66,101]
Alkyl chains with incorporated polar groups	medium	low	long	high for polymer-based adsorbents	sequence isomers, shortmers, metabolites	[13,39,40,41,42,44,45,46,47,48,49,53,56,57,63,64,73,74,101]
Aromatics	high	high	medium	high, but just for unmodified OGNs; low for ASO	sequence isomers, ASO, shortmers, longmers, metabolites	[9,10,35,43,53,56,62,63,64,65,66,67,68,69,70]
Pentafluorophenyl	medium	medium	short	-	sequence isomers, ASO shortmers, longmers, metabolites	[53,56,63]
Cholesterol	medium	low	short	-	sequence isomers, shortmers	[57,71]
Alkyl chain with quaternary nitrogen	high	medium	long	usually high, depend on OGNs modification	homooligonucleotides, shortmers, longmers, phosphorothioate OGNs diastereoisomers, ASO	[17,19,20,33,75,77,78,79,80,82,83]
Diethylaminoethyl	medium	medium		very high	sequence isomers, ASO, shortmers	[18,24,25,35,36,51,52,54,69,70,84,94,103]
Silica	low or even irreversible adsorption	low	long	medium	homooligonucleotides, shortmers, longmers,	[22,23,25,30,87,88,89,90,91]
Amide	very high	very high	medium	-	homooligonucleotides, sequence isomers, shortmers, ASO	[81,88,89,90,93,94]
Zwitterionic	high	very high	short	-	homooligonucleotides, shortmers, metabolites, ASO	[91,93]
Diol	good	high but just for polymer-based supports	long	-	homooligonucleotides, sequence isomers, shortmers	[22,23,87,88,90,91]
Oligonucleotides	good	very high	short	very high	unmodified OGNs and ASO, which are complementary to the OGN strand at the surface	[33,35,36,96,97,98,99,100,105,106,107]
